# Exploring the influence of nasal morbidity on quality of life following endoscopic endonasal skull base surgery: a retrospective cohort study of 95 patients

**DOI:** 10.1007/s10143-023-02240-9

**Published:** 2023-12-16

**Authors:** Rutwik Hegde, Vlad Prodan, Karolina Futera, Iain Hathorn, Rohit Gohil, Mark A. Hughes

**Affiliations:** 1 University of Edinburgh School of Medicine, 47 Little France Cres, Edinburgh, EH16 4TJ UK; 2 Department of Otolaryngology, Lauriston Building, Lauriston Place, Edinburgh, EH3 9EN UK; 3 Department of Clinical Neurosciences, BioQuarter, 50 Little France Crescent, Edinburgh, EH16 4SA UK

**Keywords:** Endoscopy, Skull base, Nasal morbidity, Quality of life

## Abstract

The endoscopic endonasal approach is more disruptive to normal anatomy (particularly nasal mucosa) than the transseptal submucosal microscopic approach. This may result in greater postoperative nasal morbidity, in turn reducing quality of life. We aimed to assess the severity and time course of nasal morbidity, and its impact on quality of life, following endoscopic endonasal skull base surgery in this retrospective cohort study. We identified 95 patients who underwent endoscopic endonasal skull base surgery for anterior skull base pathologies. Nasal-specific questions from the Sino-Nasal Outcome Test-22 (SNOT-22) and the Anterior Skull Base inventory (ASB-12) were combined with quality-of-life questions. Patient demographics, diagnosis, and operative data were collected from electronic records. Age of the cohort ranged from 14–83 years. Time elapsed since surgery ranged from 3–85 months. 85/95 (89%) felt that nasal morbidity associated with surgery was acceptable, given the underlying reason for, and outcome of surgery; 10/95 (11%) did not. 71/95 (75%) reported no change or improvement in olfaction 3-months following surgery. 24/95 (25%) reported a deterioration in olfaction which was mild in 7%, moderate in 7%, and severe in 11%. Nasal crusting, nasal obstruction, and headache were *moderately* problematic symptoms but improved significantly by 3-month follow-up. Nasal discharge, nasal pain, and nasal whistling were *mildly* problematic and improved significantly by 3-months. 62/95 (65%) patients reported ‘no change’ in day-to-day activities due to the effects on their nose after surgery. 19/95 (20%) had ‘mild inconvenience’, 8/95 (8%) ‘moderate inconvenience’ and 6/95 (6%) ‘severe inconvenience’. Endoscopic anterior skull base surgery is associated with nasal morbidity. Whilst 35% of patients appreciate a consequent negative impact on day-to-day life, the overwhelming majority feel that nasal morbidity is acceptable, given the wider surgical goals.

## Introduction

Endoscopic or endoscope-assisted approaches to the skull base are proliferating. Endoscopic approaches to the anterior skull base occur predominantly via the nasal corridor and paranasal sinuses [19]. In many settings, the endoscope has replaced the previously used microscopic transseptal submucosal approach [7]. There is consensus that the endoscope provides better visualisation [13] and some data suggests shorter hospital stays [10] and improved extent of tumour resection [1, 13]. However, compared with the submuco-perichondrial corridor used in microscopic surgery, the endoscopic approach is more disruptive to normal anatomy (particularly nasal mucosa) [4]. This may result in higher levels of postoperative nasal morbidity which may in turn reduce quality of life.

Multiple tools and scoring systems have been used to assess nasal morbidity after endoscopic endonasal surgery. The most common is the Sino-Nasal Outcome Test-22 (SNOT-22), a symptom-based patient-reported questionnaire consisting of 22 items reported across 5 domains (rhinologic, extra-nasal rhinologic, ear/facial, psychological/sleep). Each question is scored on a Likert scale with higher scores indicating higher levels of patient-reported nasal morbidity [6]. Importantly, the SNOT-22 questionnaire was originally developed for use in chronic rhinosinusitis and *not* in the context of endonasal endoscopic skull base surgery [2, 11]. By contrast, the Anterior Skull Base Nasal inventory-12 (ASK-12) was developed and validated specifically for endoscopic endonasal skull base surgery [12]. However, both tools are symptom-specific and do not capture the wider impact that nasal morbidity may have upon overall quality of life. As a result, other validated quality of life assessment tools (not specific to nasal morbidity) have sometimes been used separately or in parallel. For example, the 36-Item Short Form Survey (SF-36).

Appreciating the relative contribution of postoperative nasal morbidity to overall quality of life demands nuanced patient-reported outcome measures. To explore this further, we developed a hybrid patient-reported assessment tool encapsulating relevant nasal symptoms and broader impacts on quality of life. We deployed this assessment retrospectively to assess 95 patients who underwent endoscopic endonasal skull base surgery during the period in which our unit transitioned from microscopic to endoscopic approaches (2014 to 2021).

## Materials and methods

All patients who underwent endoscopic endonasal skull base surgery in Edinburgh between October 2014 and November 2021 were identified from a prospectively maintained, Caldicott-approved database. During this transition period, from a microscopic to predominantly endoscopic endonasal approach, 146 patients underwent endoscopic anterior skull base surgery. Of these, 12 patients were deceased by the time of the study, 28 could not be contacted, and 11 patients declined to participate. 95 patients (52 female and 43 male, range of time since operation 3 to 85 months) were contacted and completed a telephone questionnaire.

Patient demographic data, underlying diagnosis, and surgical operative data were collected from our electronic patient management system. Pre-operative data included patient age at the time of operation, sex, pathology, previous transsphenoidal surgery (number of previous surgeries and whether endoscopic or microscopic approach), and any pertinent past medical history.

Patients completed the questionnaire (detailed below) retrospectively and by telephone. For patients who underwent more than one transsphenoidal surgery during the study time window, the questionnaire was performed only once and in relation to their most recent surgery. Data was consolidated in Excel (Microsoft, USA) and statistical analysis (t-test) was performed using GraphPad Prism (Dotmatics, USA). Since the pathology being targeted by surgery may itself impact nasal morbidity and determines specific aspects of the surgical approach which variably impact nasal morbidity, we analysed separate subgroups: (1) non-expanded endoscopic transsphenoidal approaches to the sella, (2) expanded transsphenoidal approaches to the sella, (3) approaches to nasal sinus-centred pathology, and (4) operations for olfactory neuroblastoma (Table 1).

**Table 1:** Hybrid patient-reported questionnaire

Do you feel that the after-effects on your nose following surgery are acceptable, given the reason you had your surgery?	Yes	No	Unsure	Free text response	-	-
How have effects on your nose following surgery affected your day-to-day activities?	No effect (1)	Mild inconvenience requiring no intervention-on (2)	Moderate inconvenience requiring ongoing nasal care (3)	Severe inconvenience affecting you day and night (4)	Free text response	-
Crusting	No problem (1)	Mild problem (2)	Moderate problem (3)	Severe problem (4)	Very severe problem (5)	Duration
Thick discharge	No problem (1)	Mild problem (2)	Moderate problem (3)	Severe problem (4)	Very severe problem (5)	Duration
Nasal pain	No problem (1)	Mild problem (2)	Moderate problem (3)	Severe problem (4)	Very severe problem (5)	Duration
Nasal obstruction	No problem (1)	Mild problem (2)	Moderate problem (3)	Severe problem (4)	Very severe problem (5)	Duration
Headache	No problem (1)	Mild problem (2)	Moderate problem (3)	Severe problem (4)	Very severe problem (5)	Duration
Nasal whistling	No problem (1)	Mild problem (2)	Moderate problem (3)	Severe problem (4)	Very severe problem (5)	Duration
Difficulty obtaining rest due to nasal symptoms	No problem (1)	Mild problem (2)	Moderate problem (3)	Severe problem (4)	Very severe problem (5)	-
Sense of smell at 3-months post-op	Severely worse (1)	Moderately worse (2)	Mildly worse (3)	No change (4)	Improv-ement (5)	-

## Results

The age of the cohort ranged from 14 to 83 years (mean 51). Time elapsed since surgery ranged from 3 to 85 months (mean 34 months). 48 patients underwent surgery for non-functional pituitary macroadenoma and 13 for functional adenomas (8 for acromegaly, 4 for Cushing’s disease, and 1 prolactinoma). 9 patients underwent surgery for craniopharyngioma, 6 for Rathke’s cleft cyst, 6 for CSF leak (due to encephalocoele or trauma), 3 for olfactory neuroblastoma (all of whom underwent a concurrent transcranial subfrontal approach), 2 for clival chordoma, 2 for chondrosarcoma. There was one instance of surgery for each of meningioma, trigeminal schwannoma, sinonasal sarcoma, juvenile nasal angiofibroma, osteoma, and sellar arachnoid cyst.

Considering the cohort together, 85/95 (89%) felt that the nasal morbidity associated with surgery was acceptable to them, given the underlying reason and outcome of surgery; 10/95 (11%) did not. 62/95 (65%) patients reported ‘no change’ to day-to-day activities due to effects on their nose after surgery. 19/95 (20%) had ‘mild inconvenience’, 8/95 (8%) ‘moderate inconvenience’ and 6/95 (6%) reported ‘severe inconvenience’.

**Fig. 1:**
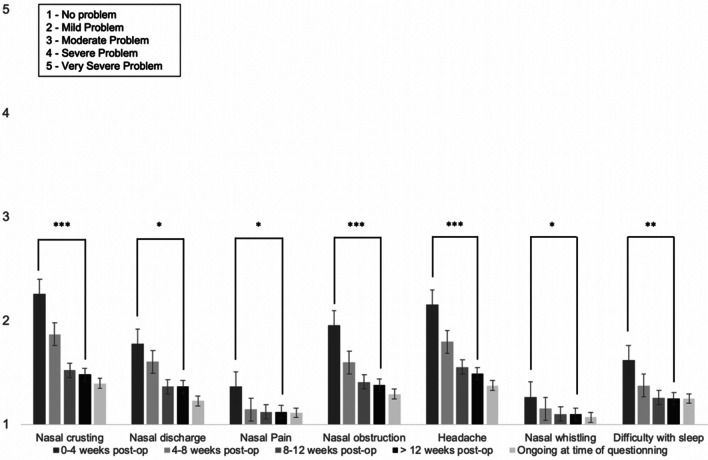
Mean nasal symptoms severity (Likert score 1–5), and their duration after surgery, for the entire cohort of 95 patients

71/95 (75%) reported no change or improvement in olfaction following surgery. 24/95 (25%) reported a deterioration in olfaction at the time of questioning: mild in 7%, moderate in 7%, and severe in 11%. Nasal crusting, nasal obstruction, and headache were moderately problematic symptoms initially, but all improved significantly by 3-month follow-up. Nasal discharge, nasal pain, and nasal whistling were mildly problematic issues which also improved significantly by 3-month follow up. See Fig. 1.

### Non-expanded endoscopic transsphenoidal approaches to the sella (*n* = 68, including pituitary adenoma, Rathke cleft cyst)

59/68 (87%) felt that the after-effects of surgery were acceptable, given the underlying reason and outcome of surgery. One patient was not sure and 8/69 (12%) did not feel that the nasal morbidity associated with surgery was acceptable. Free text responses included: nasal symptoms were “*initially very bad but improved*”, nasal symptoms “*most definitely*” acceptable, “*absolutely*” acceptable, ‘*no problems after surgery*”, “*slight inconvenience*”, “*tumour was removed, so it’s all good*”, surgery “*was essential as tumour was affecting my eyesight*”, surgery was “*definitely worth it because I would have lost my eyesight*”, “*it was horrendous initially but it did get better*”, “*it was the right thing to do, I was aware of possible symptoms*”, “*everyone was great but idk (sic) if it was worth it. I don’t feel great, very tired now*”.

**Fig. 2:**
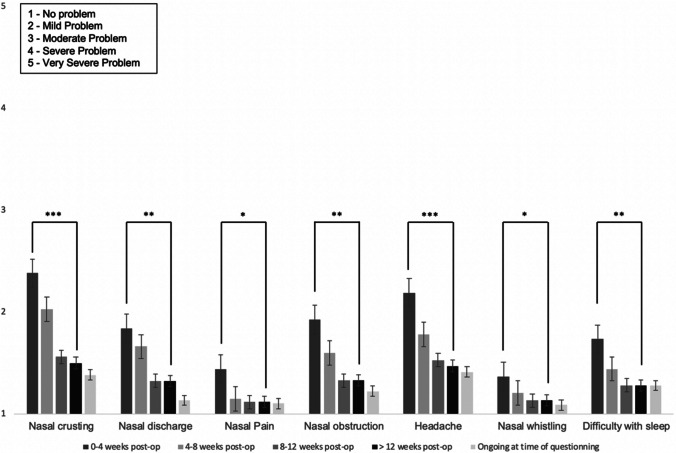
Mean nasal symptoms severity (Likert score 1–5) and their duration after surgery, for patients undergoing non-expanded endoscopic transsphenoidal surgery

For 25/68 patients (37%), the impact of surgery on their nose continues to cause some degree of inconvenience. For 13/68 (19%) this is mild, for 8/68 (12%) this is moderate, and for 4/68 (6%) patients, the inconvenience is severe. 50/68 (74%) reported no change or improvement in olfaction following surgery. 18/68 patients (26%) reported a deterioration in olfaction: mild in 9%, moderate in 10%, and severe in 7%. Figure 2 shows the impact of surgery on nasal symptoms of crusting, discharge, pain, obstruction, headache, whistling, and capacity to achieve rest. Headache and nasal crusting were the most problematic of these symptoms (of mild/moderate severity). The severity of all nasal symptoms reduced significantly by 3-month follow-up.

### Expanded transsphenoidal approaches to the sella (*n* = 15, as was indicated for surgery for chordoma, craniopharyngioma, meningioma)

**Fig. 3:**
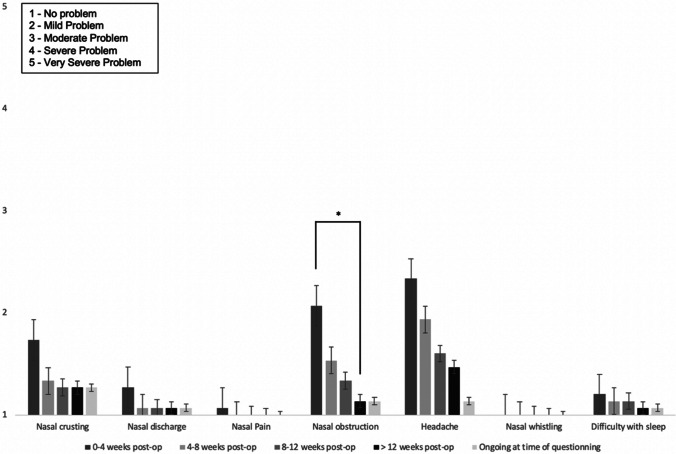
Mean nasal symptoms severity (Likert score 1–5), and their duration after surgery, for patients undergoing expanded endoscopic transsphenoidal surgery

14/15 (93%) felt that the after-effects of surgery were acceptable, given the underlying reason and outcome of surgery. 1/15 (7%) did not feel that nasal morbidity associated with surgery was acceptable. Free text responses included: nasal side effects were “*absolutely acceptable*”, and they are “*acceptable if able to remove the tumour*”. 12/15 (80%) reported no change or improvement in olfaction following surgery. 3/15 patients (20%) reported a deterioration in olfaction: mild in 7% and severe in 13%. Figure 3 shows the impact of surgery on the nasal symptoms of crusting, discharge, pain, obstruction, headache, whistling, and capacity to achieve rest. Nasal obstruction and headache were mildly problematic in the early post-operative period but improved by 3-months. For 4/15 patients (27%), nasal side effects continue to cause some degree of inconvenience. For 3/15 this is mild and for 1/15 patients this is severe inconvenience.

Considering those patients who underwent revision transsphenoidal surgery, there was a similar impact on olfaction when compared with those undergoing index procedures. 13 patients underwent revision operations. 11/13 (85%) reporting stable or improved olfaction post-surgery and 2/13 reporting deterioration (one mild, one severe).

### Endoscopic approaches to nasal sinus-centred pathology and the paramedian floor of the anterior fossa (*n* = 9, including anterior fossa encephalocele and sinonasal tumours)

**Fig. 4:**
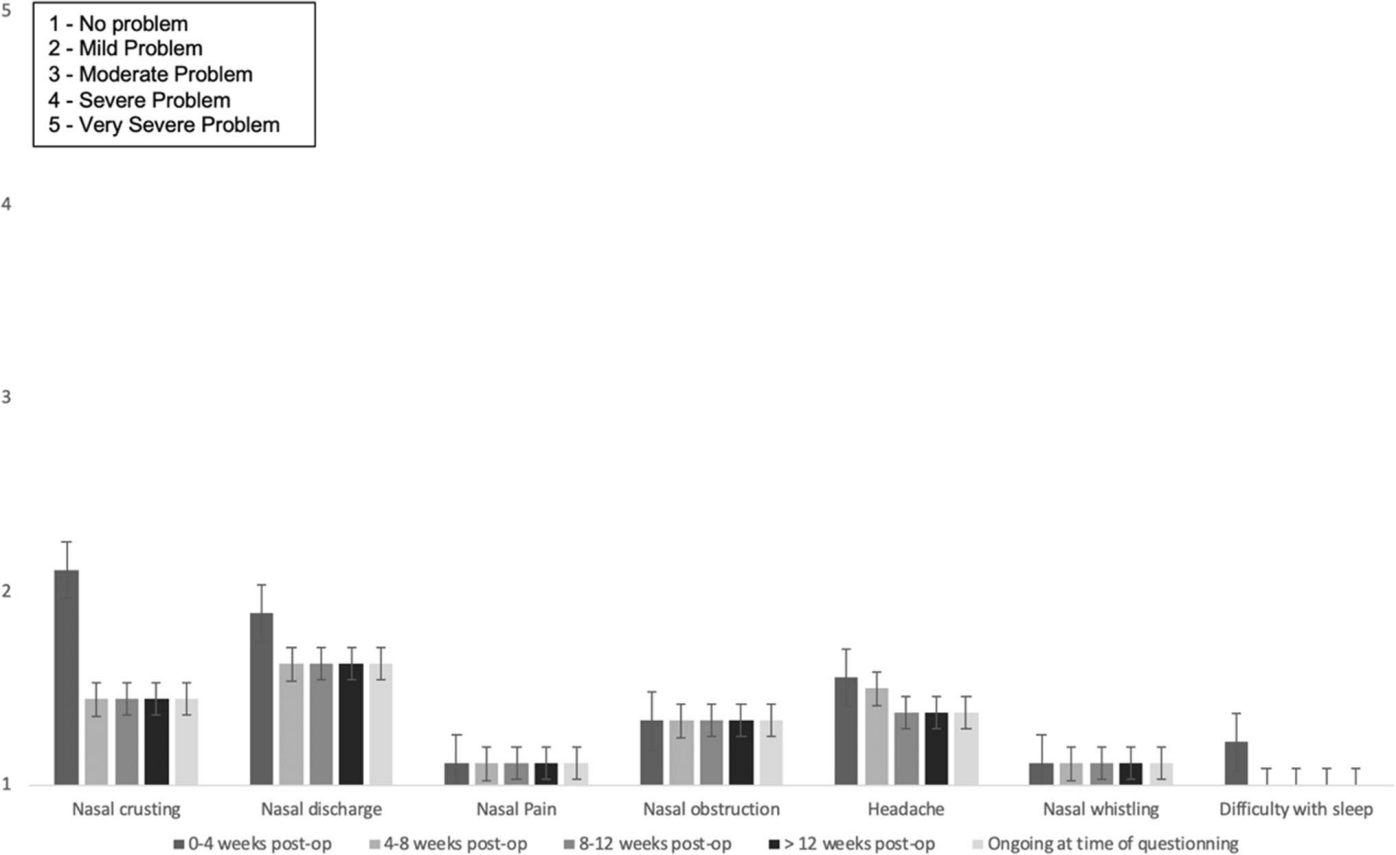
Mean nasal symptoms severity (Likert score 1–5), and their duration after surgery, for patients undergoing surgery for pathologies centred in the nose or paramedian floor of the anterior fossa

9/9 (100%) felt that the after-effects of surgery were acceptable, given the underlying reason and outcome of surgery. Free text responses included: “*nose symptoms now are not as bad as before*”. 9/9 (100%) reported no change or improvement in olfaction following surgery. Figure 4 shows the impact of surgery on nasal symptoms of crusting, discharge, pain, obstruction, headache, whistling, and capacity to achieve rest. In this cohort, these symptoms were mild at worst and often absent. For 3/9 patients (33%), the impact on their nose caused a mild degree of inconvenience.

### Surgery for olfactory neuroblastoma (*n* = 3) – all underwent combined endonasal and transcranial subfrontal approach

**Fig. 5:**
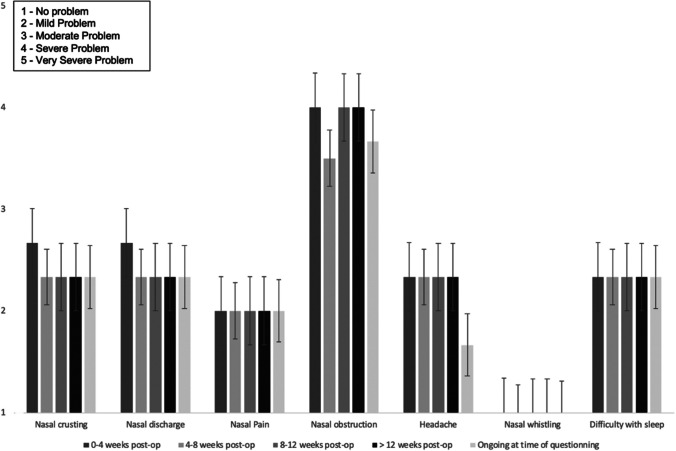
Mean nasal symptoms severity (Likert score 1–5), and their duration after surgery, for three patients undergoing a combined endonasal and transcranial subfrontal approach to manage olfactory neuroblastoma

3/3 (100%) felt that the after-effects of surgery were acceptable, given the underlying reason and outcome of surgery. Free text responses included: nasal side effects were a “*small price to pay*” and nasal side effects were “*absolutely acceptable*”. This pathology directly threatens olfaction, and all three cases were treated by combined endonasal and transcranial sub-frontal approaches. All three patients had subjectively severely worse olfaction following treatment. Figure 5 shows the impact of surgery on nasal symptoms of crusting, discharge, pain, obstruction, headache, whistling, and capacity to achieve rest. Nasal obstruction was a severe problem whilst nasal crusting and nasal discharge were moderately severe. One patient reports severe inconvenience because of nasal morbidity.

## Discussion

A meta-analysis in 2019 by Bhenswala et al. assessed sino-nasal morbidity after endoscopic endonasal skull base surgery [2]. 19 studies were analysed, combining 27 datasets into a population of 1025 patients and reporting pre-operative and follow-up SNOT-22 data at < 4 weeks, 12-weeks, 26-weeks, 52-weeks, and > 96-weeks follow-up. Overall, SNOT-22 scores were significantly worse at the < 4-week follow-up, but significantly improved at all subsequent follow-up times. Whilst this work illustrates that this scoring system captures important symptomatic changes, it does not correlate these changes with the overall impact on patient-reported quality of life, nor relate the impact to the underlying disease process being targeted by the operation. We qualitatively assessed relevant studies published since Bhenswala’s meta-analysis of 2019, reviewing the Sino-Nasal Outcomes Test-22 (SNOT-22) and Anterior Skull Base Inventory-12 (ASB-12) and comparing – when possible – with quality-of-life assessment tools (SF-36, ASBS-Q, and the EQ-5D). Embase and MEDLINE databases were interrogated with the search phrase [(“endoscopic endonasal” OR “endoscopic transsphenoidal”) AND (“nasal morbidity” OR “sino-nasal outcome” OR “sino-nasal outcome test 22”)]. 12 contemporary papers were identified [3, 5, 8, 9, 14–21]. Scagnelli et al. demonstrated that overall SNOT-22 scores followed the expected rise and fall pattern after endoscopic transsphenoidal surgery. However, when looking at subdomains, only the rhinologic and sleep domains were found to be significantly different [18]. Hallén et al. found similar results with only rhinological domains (taste and smell; postnasal discharge; thick nasal discharge; and need to blow nose scores) significantly worsening [8]. Novák et al. found a similar pattern. The need to blow the nose, nasal discharge, thick discharge, and loss of smell and taste were all significantly worse, despite no significant difference in the overall score being noted [14]. Hannan et al. found that the nasal domains and olfactory score were significantly worse but found no significant change in the overall score [9]. This shows again that the SNOT-22 is a valid method of detecting sinonasal symptom change after endonasal skull base surgery but reiterates that it lacks specificity, due to several unnecessary and non-relevant components (e.g., assessing ear fullness, ear pain, and dizziness).

Regarding quality of life, Schreiber et al. found that while there was no significant difference between preoperative and 6-month follow-up in either the SNOT-22 or ASK-12 scores, there *was* a significant improvement in the SF-36 quality of life score [19]. Dolci et al. also found no significant difference in SNOT-22 scores at 3- and 6-months follow-up but did find a significant improvement in SF-36 at both time points [5]. Neiderman et al. found an improvement on the ASBS-Q at 4 to 6 months in the “pain-related” and “vitality-related” domains in the absence of a significant difference in SNOT-22 [3]. Hallén et al. noted a significant worsening of the rhinologic component of the SNOT-22 at 6-months compared with baseline, yet a significant *increase* on the EQ-5D [8]. These changes are therefore likely to reflect other aspects of the patient journey. For example, improvement in vision after decompressing the optic apparatus might well outweigh, in the patient’s opinion, mildly inconvenient post-operative sinonasal symptoms.

We found that whilst 85/95 (89%) felt that the nasal morbidity associated with surgery was acceptable, given the underlying reason and outcome of surgery, 10/95 (11%) did not. It is important to consider this minority in more detail. Some specific cases are considered below:

(i) A 57-year-old chef, with no significant past medical history, had a non-secretory pituitary macro-adenoma causing optic apparatus compression. Post-operatively, he reported moderate nasal crusting, mild nasal discharge, no nasal pain, mild ongoing nasal obstruction, no headache, ongoing nasal whistling, pre-existing, and ongoing issues with sleep, and reported that his smell was significantly worse. His vision recovered well. He feels ‘constantly tired’ after surgery, though he has normal postoperative pituitary function. He disengaged from follow-up from the outset, with numerous failures to attend for follow-up imaging, nursing, endocrine, and neurosurgical assessments. As a chef, the impact of impaired olfaction will be particularly pertinent.
(ii) A 40-year-old woman had a diagnosis of acromegaly. Her past medical history was significant for ongoing opiate addiction, anxiety and depression, and prior surgery for cauda equina syndrome. Post-operatively, she reported severe nasal crusting, discharge, and mild obstruction (all of which resolved within a month). She has ongoing headaches, severe problems with sleep, and reports that her sense of smell is mildly worse. Whilst her growth hormone excess was significantly improved by surgery, she did quite meet criteria for remission. She is currently on medical therapy (a somatostatin analogue) and there is no surgically accessible residual adenoma on post-operative MRI. Whilst the nasal symptoms clearly contribute to her dissatisfaction, her overall quality of life is impacted by other factors. These include those related to the underlying diagnosis of acromegaly and are probably compounded by her comorbidities.
(iii) A 59-year-old woman underwent a 5^th^ operation for a recurrent Rathke’s cleft cyst causing compressive optic neuropathy and visual loss. Her four prior operations were microscopic. She has a past medical history of obsessive-compulsive disorder, depression, anxiety, delusional disorder, obesity, and panhypopituitarism. Post-operatively, she appreciated an immediate improvement in vision, no change to smell, and only transient moderate nasal obstruction. The most recent operation involved minimal re-opening of an existing sphenoidotomy and no disturbance of the turbinates. Despite improvement in her vision, and only very subtle surgery in the nose, she did not feel nasal morbidity was acceptable. It is likely that her extensive psychiatric comorbidities inform her own overall assessment.
(iv) A 69-year-old woman underwent revision surgery for a recurrent non-secretory pituitary macroadenoma causing compressive optic neuropathy. Her past medical history included osteopenia, vertebral insufficiency fractures, and hypopituitarism (following prior transsphenoidal operations). Post-operatively, her vision was well-preserved. She reported no nasal side-effects other than mildly reduced sense of smell. She has since gone on to have postoperative radiotherapy with significant ongoing fatigue. The fatigue seems to be clouding her overall quality of life assessment, leading to global dissatisfaction.

These cases highlight the difficulty in trying to define and quantify the relative contribution of postoperative nasal symptoms to *overall* quality life. Some patients did not feel surgery was worthwhile despite minimal nasal side-effects and clear success in achieving the primary surgical goal (e.g., preservation of vision). Some reported significant nasal symptoms but found this entirely acceptable, given other outcomes from surgery. Some patients have separate health or social issues which dominate or cloud their own assessment of the success – or otherwise – of their endoscopic skull base surgery. As such, it may be impossible to alter or improve some of the issues at play.

These findings echo those of some prior studies, where quality of life changes were not congruous with sinonasal symptom burden [5, 19]. Interestingly, patients undergoing some of the most expanded (and therefore most morbid) surgical approaches (e.g., for olfactory neuroblastoma or chordoma) had relatively high levels of satisfaction. These tumours are not benign (compared with pituitary adenoma or Rathke cleft cyst) and the way we counsel patients and articulate surgical goals in these contexts is consequently very different. This will colour how patients consider post-operative morbidity and may explain the relative tolerance of nasal morbidity.

We now provide more granular detail during consent regarding potential nasal morbidity from anterior endoscopic skull base surgery, informed by data from this study. From a technical perspective, we try to avoid resection of nasal turbinates unless necessary for access. We underline the utility of and strongly encourage post-operative nasal douching (from 48 h). We also review patients earlier in the ENT clinic for nasoendoscopy and, when needed, decrusting.

This study has limitations, notably its retrospective nature. The variability in time of follow up is suboptimal, ranging from 3 to 85 months. Patients that were followed up very recently after surgery may still be in the recovery period and report symptoms which may yet resolve. Conversely, patients that were followed up several years after surgery may have difficulty in accurately remembering symptoms and their duration leading to recall bias. Future studies would be improved by conducting the questionnaire at set postoperative time points, including assessment of pre-operative sinonasal symptoms.

## Conclusions

Endoscopic anterior skull base surgery is associated with nasal morbidity. For the overwhelming majority of patients, nasal morbidity is acceptable given the underlying context and outcome of surgery. Nasal crusting, nasal obstruction, and headache are moderately problematic symptoms initially but improve significantly by 3-month follow-up. Nasal discharge, nasal pain, and nasal whistling are mildly problematic issues and improve significantly by 3-month follow-up. A small minority of patients appreciate a significant deterioration in quality of life which they attribute to postoperative nasal morbidity. Optimisation of consent, surgical technique, and follow-up are all important in minimising morbidity and managing expectations.

## Data Availability

All data that was used in the study to derive our findings is available upon request from the corresponding author.
